# Long-term results of oversized balloon dilation for benign anastomotic biliary strictures: initial two-center experience

**DOI:** 10.1590/0100-3984.2021.0027

**Published:** 2022

**Authors:** Thiago Franchi Nunes, Riccardo Inchingolo, Reinaldo Morais Neto, Tiago Kojun Tibana, Vinicius Adami Vayego Fornazari, Joaquim Maurício da Motta-Leal-Filho, Stavros Spiliopoulos

**Affiliations:** 1 Hospital Universitário Maria Aparecida Pedrossian da Universidade Federal de Mato Grosso do Sul (HUMAP-UFMS), Campo Grande, MS, Brazil.; 2 Division of Interventional Radiology, Department of Radiology, Madonna delle Grazie Hospital, Matera, Italy.; 3 Escola Paulista de Medicina da Universidade Federal de São Paulo (EPM-Unifesp), São Paulo, SP, Brazil.; 4 Instituto do Câncer do Estado de São Paulo (Icesp) and Instituto do Coração do Hospital das Clínicas da Faculdade de Medicina da Universidade de São Paulo (InCor/HC-FMUSP), São Paulo, SP, Brazil.; 5 Second Department of Radiology, Interventional Radiology Unit, National and Kapodistrian University of Athens, Medical School, “Attikon” University Hospital, Athens, Greece.

**Keywords:** Anastomosis, surgical/adverse effects, Dilatation/methods, Bile ducts/physiopathology, Constriction, pathologic/etiology, Magnetic resonance imaging, Tomography, X-ray computed, Anastomose cirúrgica/efeitos adversos, Dilatação/métodos, Ductos biliares/fisiopatologia, Constrição patológica/etiologia, Ressonância magnética, Tomografia computadorizada

## Abstract

**Objective:**

To describe, assess the feasibility of, and quantify the long-term patency achieved with percutaneous transhepatic biliary dilation using the anastomotic biliary stricture (ABS) oversized balloon dilation technique as a single-step procedure for the treatment of benign anastomotic biliary strictures following hepatobiliary surgery.

**Materials and Methods:**

This was a retrospective, two-center study including 16 consecutive cases of symptomatic benign biliary-enteric strictures. After assessment of the diameter of the bile duct by computed tomography or magnetic resonance imaging, the strictures were dilated with oversized balloons (40-50% larger than the bile duct diameter) and an external biliary-enteric drain was placed. After drain removal, clinical symptoms and laboratory test results were evaluated every three months, whereas follow-up magnetic resonance imaging was performed at 30 days out and follow-up computed tomography was performed at 6 and 12 months out.

**Results:**

The mean follow-up time was 31.8 ± 8.15 months. Kaplan-Meier-estimated 1-, 2-, and 3-year patency rates were 88.2%, 82.4%, and 82.4%, respectively. There was one major complication—a small dehiscence of the anastomosis—which extended the catheter dwell time. Minor complications occurred in two cases—one small perihepatic hematoma and one segmental thrombosis of the left portal branch—neither of which required further intervention.

**Conclusion:**

The single-step ABS oversized balloon dilation technique is a feasible treatment for benign anastomotic biliary-enteric strictures. The technique appears to be associated with high rates of long-term clinical success and patency.

## Abstract (pt)

**Objetivo:**

Descrever o procedimento, avaliar a viabilidade e perviedade em longo prazo da dilatação biliar trans-hepática percutânea usando a técnica de dilatação por balão superdimensionado para o tratamento em uma única etapa de estenose biliar anastomótica benigna após cirurgia hepatobiliar.

**Materiais e Métodos:**

Este estudo retrospectivo de dois centros incluiu 16 casos consecutivos de estenoses bilioentéricas benignas sintomáticas. A dilatação das estenoses com superdimensionamento do balão de 40-50% foi realizada após avaliação pré-procedimento do diâmetro do ducto biliar por tomografia computadorizada ou ressonância magnética e um dreno externo foi colocado. Os sintomas clínicos e exames laboratoriais foram avaliados a cada três meses após a remoção do dreno, enquanto o acompanhamento radiológico foi realizado com ressonância magnética em 30 dias e tomografia computadorizada em 6 e 12 meses.

**Resultados:**

O tempo médio de seguimento foi de 31,8 ± 8,15 meses. As estimativas de perviedade em um, dois e três anos foram 88,2%, 82,4% e 82,4%; respectivamente. Houve uma complicação importante, com pequena deiscência da anastomose biliodigestiva, que exigiu prolongamento do tempo de permanência do dreno externo. Complicações menores ocorreram em dois casos, um pequeno hematoma peri-hepático e uma trombose segmentar do ramo portal esquerdo e nenhum deles necessitou de intervenção adicional.

**Conclusão:**

A técnica de dilatação com balão superdimensionado para o tratamento de estenoses biliares anastomóticas benignas foi viável para o tratamento de estenoses anastomóticas bilioentéricas benignas. A técnica parece estar associada a altas taxas de perviedade e de sucesso clínico no longo prazo.

## INTRODUCTION

Benign anastomotic biliary-enteric stricture is one of the main and most severe complications of hepatobiliary surgery. The incidence of such stricture after hepaticojejunostomy ranges from 2.6% to 24.0%, and the condition is usually caused by fibrosis and scarring^(1-3)^. Treatment options for benign postoperative anastomotic biliary-enteric strictures include revision surgery, endoscopic treatment, and transhepatic percutaneous treatment.

In revision surgery for anastomotic biliary-enteric strictures, there are technical difficulties due to the shortening of the remaining bile duct, inflammation, adhesions in the abdominal cavity, and comorbidities. Endoscopic access to the stricture may be hindered by altered intestinal anatomy in the postoperative period, often due to diversion of the intestinal transit with “Roux-en-Y” reconstructions. Therefore, patients with such strictures are frequently referred to interventional radiology for percutaneous transhepatic treatment and some will undergo multiple complementary procedures and will be hospitalized multiple times^(1-4)^. Although percutaneous transhepatic biliary drainage (PTBD) is a well-known technique, there are currently few data regarding a single-step dilation technique using oversized balloon catheters and short-term biliary drainage.

This study aims to describe, assess the safety of, and determine the feasibility of, as well as to quantify the long-term patency achieved with the anastomotic biliary stricture (ABS) oversized balloon dilation technique for the management of benign anastomotic biliary-enteric strictures.

## MATERIALS AND METHODS

### Study design and patient selection

This was a retrospective study carried out at two hospitals where the ABS oversized balloon dilation technique is practiced. A meticulous search of the databases of the two hospitals, limited to the period from January 2016 to December 2018, revealed that 16 patients had undergone hepatobiliary surgery and subsequently developed benign anastomotic biliary-enteric stricture that was refractory to endoscopic treatment. The strictures were identified by computed tomography (CT), magnetic resonance (MR) cholangiography, or both, in conjunction with laboratory test results and symptoms consistent with the condition (elevated liver enzyme levels, cholangitis, and jaundice). Patients were selected for treatment with the ABS oversized balloon dilation technique if they had a benign stricture documented on CT or MR cholangiography and had undergone unsuccessful endoscopic treatment. Patients with malignant strictures were excluded, as were those who had previously been treated for benign strictures after biliary-enteric surgery. The study was approved by the local institutional review board. Because of the retrospective nature of the study, the requirement for written informed consent to be included in the study was waived. However, all of the patients had been informed of the possibilities of complications associated with the ABS oversized balloon dilation technique (e.g., stricture recurrence, stone formation, and bowel obstruction) and had given written informed consent to undergo the procedure.

### Description of the technique

Each procedure was performed by one of two interventional radiologists with 8 and 10 years of experience in hepatobiliary procedures, respectively. A broad-spectrum antibiotic (ciprofloxacin, 400 mg) was administered intravenously 6 h before each procedure. All of the procedures were performed under conscious sedation (with 3-5 mg of midazolam and 50-75 mg of pethidine hydrochloride) with a local anesthetic (2% lidocaine).

**Figure 1:**
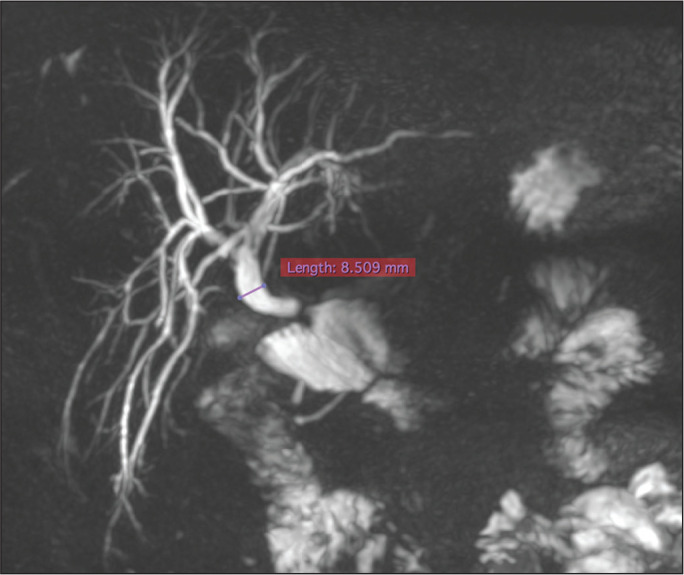
MR cholangiography, in an oblique maximum intensity projection acquisition, of a patient with biliary-enteric anastomosis stricture nine months after video cholecystectomy for acute calculous cholecystitis. Maximum diameter of the common bile duct, 8.5 mm.

**Figure 2:**
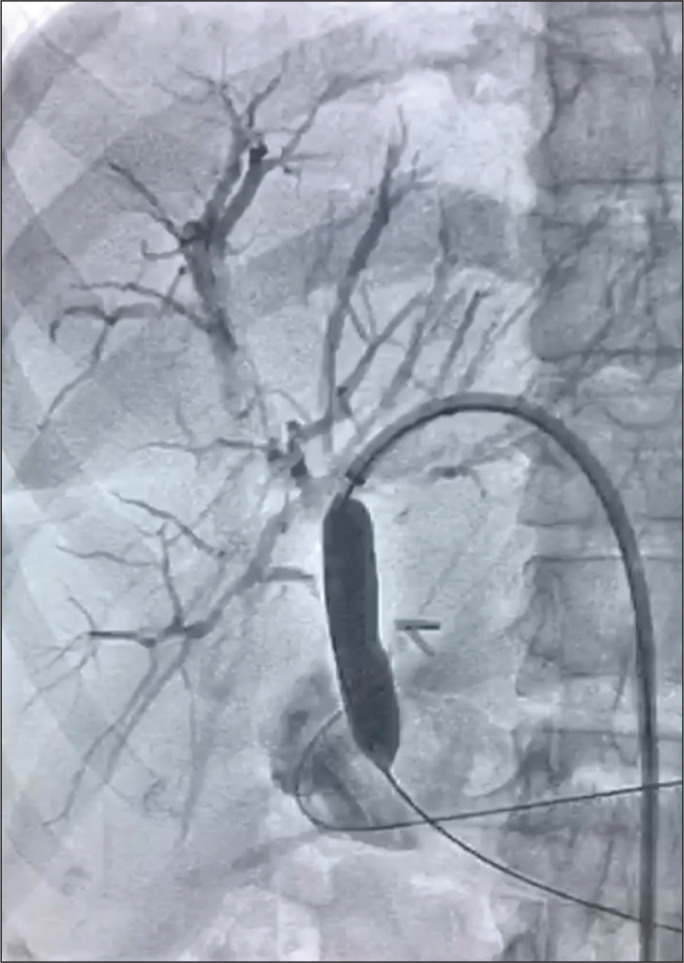
Dilation using a 12 × 80 mm balloon at the point of biliary-enteric stricture.

**Figure 3:**
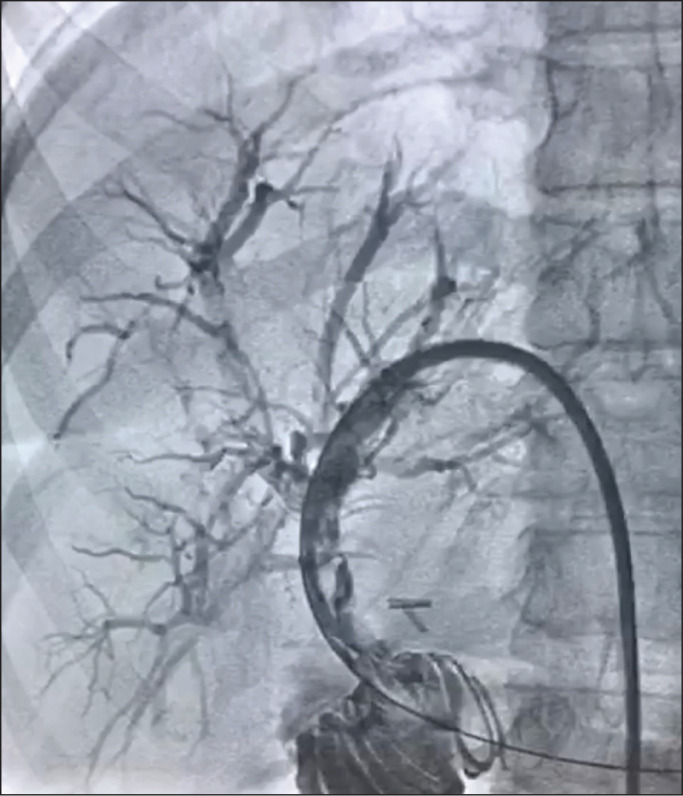
MR cholangiography after two cycles of dilation, showing a satisfactory result and no residual stenosis.

The anastomotic stricture was dilated with a noncompliant balloon catheter that had a diameter of 10, 12, 14, or 16 mm (Athletis; Boston Scientific), in accordance with the diameter of the affected bile duct, as determined on a coronal CT or MR cholangiography scan. The diameter of the bile duct was measured at approximately 1 cm proximal to the obstruction (Figure 1) and visualized with the OsiriX viewer, version 11.0 (Pixmeo SARL, Bernex, Switzerland). The balloon diameter chosen was 40-50% larger than the bile duct diameter prior to the development of the stricture. The balloon catheter was always inflated twice (Figure 2). During the first inflation, the dilation was continued until no residual lesion “waist” was noted and the balloon was kept inflated for 10 min. The duration of the second inflation was 5 min. In patients with bifurcated strictures, the contralateral stricture was crossed using the same, initial, PTBD access and subsequent balloon dilation was performed. If lesion crossing was not technically feasible from the same access, a percutaneous access to the duct of the contralateral lobe was created in order to perform the balloon dilation. After balloon dilation, MR cholangiography was performed (Figure 3); if the passage of contrast was slow or ≥ 30% residual stricture was observed, additional 5-min balloon inflations were performed until the stricture was eliminated or reduced to < 30%. An 8-Fr pigtail catheter was placed across the anastomosis within the bowel, for drainage. Patients were discharged after 24-48 h of observation. A seven-day course of ciprofloxacin and metronidazole was prescribed for all patients, and the external drain was left in place for three to five days. If there were lacerations or dehiscence of the biliary-enteric anastomosis, the catheter dwell time was extended to at least 30 days. In all cases, forceps biopsy of the stenotic area was performed before balloon dilation, as previously described^(5)^, to rule out malignancy.

### Follow-up

Every three months after removal of the drainage catheter, clinical symptoms and laboratory test results, including blood cell counts and liver function parameters, were evaluated on an outpatient basis. At 30-40 days after the ABS oversized balloon dilation procedure, MR cholangiography was performed to identify any complications. Contrast-enhanced CT was performed at 6 and 12 months after the procedure. Any symptoms, CT findings, MR cholangiography findings, or laboratory test results suggestive of recurrence of the stricture were further investigated with percutaneous cholangiography. Additional procedures were performed as needed.

### Definitions and analyses

The primary efficacy endpoint was primary patency, defined as the interval between the first treatment cycle and recurrence of the stricture. In patients without recurrence, primary patency was assumed to be the interval between drainage catheter removal and the last follow-up evaluation. The primary safety endpoint was the rate of procedure-related complications. Complications were classified as major or minor in accordance with previously published guidelines^(6)^. Secondary endpoints were technical success, clinical success, and PTBD dwell time. Technical success was defined as patent contrast passage through a dilated anastomotic stricture with < 30% residual stricture on the final cholangiogram. Clinical success was defined as resolution of the symptoms of cholangitis and normalized liver function. Cholangitis was defined as an episode of fever, laboratory test results indicative of inflammation, and a positive bacterial bile sample^(7)^.

### Statistical analysis

Descriptive statistics were calculated as mean and standard deviation or as median and interquartile range for continuous variables, whereas they were calculated as absolute and relative frequencies for categorical variables. Continuous data were assessed with the Kolmogorov-Smirnov goodness-of-fit test to determine whether they originated from normal distributions. Unpaired Student’s t-tests were used in order to assess the significance of pre- and post-procedural laboratory test values for continuous variables that passed the normality test, whereas the Mann-Whitney test was used for those that did not. Kaplan-Meier life-table analysis was used in order to calculate primary patency. Statistical analysis was performed with the IBM SPSS Statistics software package, version 25.0 (IBM Corp., Armonk, NY, USA). The threshold of statistical significance was set at *p* ≤ 0.05.

## RESULTS

### Patients

We initially identified 23 patients who had developed benign strictures after biliary-enteric surgery during the study period. Seven patients were excluded for not meeting the study criteria: five because they had malignant strictures; and two because they had previously been treated for benign strictures due to biliary-enteric surgery. Therefore, the final sample comprised 16 patients who had undergone hepatobiliary surgery and subsequently developed benign anastomotic biliary-enteric stricture that was refractory to endoscopic treatment. The mean age was 54 years (range, 32-74 years), and the male-to-female ratio was 9:7. Of the 16 patients in the sample, one had undergone liver surgery and the rest had undergone extrahepatic surgery. Fourteen (87.7%) of the patients had undergone simple anastomosis (hepaticojejunostomy). We analyzed the underlying disease, the time from surgery to transhepatic treatment, the type of biliary-enteric anastomosis, and the relevant laboratory test results (before and 30 days after percutaneous treatment). Baseline patient characteristics are presented in Table 1.

**Table 1:** Baseline demographics and procedural details.

Variable	n (%)
Sex	
Female	7 (43.8)
Male	9 (56.2)
Type of surgery	
Extrahepatic	15 (93.8)
Hepatic	1 (6.2)
Type of biliary-enteric anastomosis	
Double right and left hepaticojejunostomy	2 (12.5)
Simple hepaticojejunostomy	14 (87.5)
Time from surgical procedure to stricture onset	
< 3 months	2 (12.5)
≥ 6 months	10 (62.5)
3-6 months	4 (25.0)
Clinical presentation	
Jaundice and pruritus	1 (6.2)
Cholangitis	7 (43.8)
Cholangitis and pruritus	2 (12.5)
Jaundice	4 (25)
Elevated liver enzymes only	2 (12.5)
Balloon size	
12 × 40 mm	1 (6.2)
14 × 40 mm	3 (18.8)
16 × 40 mm	10 (62.5)
16 × 50 mm	1 (6.2)
16 × 60 mm	1 (6.2)
External drainage time (days)	
3	2 (12.5)
4	3 (18.8)
5	6 (37.5)
6	1 (6.2)
7	3 (18.8)
21	1 (6.2)
Complication/grade (CIRSE classification)	
Biliary leak	1 (33.3)
Perihepatic hematoma	1 (33.3)
Segmental portal thrombosis	1 (33.3)
Treatment of complication	
Conservative	2 (66.7)
Prolonged drainage	1 (33.3)
Recurrence	
No	13 (81.2)
Yes	3 (18.8)
Time from procedure to recurrence	
< 12 months	2 (66.7)
≥ 12 months	1 (33.3)

CIRSE, Cardiovascular and Interventional Radiological Society of Europe.

### Outcomes of the procedure

Technical success was achieved in all cases—after a single balloon dilation session in 13 (81.2%) of the 16 patients, after two sessions in three (18.8%), and after three sessions in one (6.2%). Therefore, a total of 19 procedures were performed. Clinical success was also achieved in all cases. There was only one major complication—a small dehiscence of the biliary-enteric anastomosis, observed on a follow-up cholangiogram. In that case, it was decided that the external biliary drain would be left in place for 40 days beyond the usual seven days. In addition, the intravenous antibiotic therapy was extended by two days, which consequently extended the hospital stay. Minor complications occurred in two (10.5%) of the 19 procedures. One patient had a small perihepatic hematoma after transhepatic puncture, and another developed segmental thrombosis of the left portal branch. Conservative treatment with analgesics and bed rest was prescribed in both cases, and no clinical sequelae were noted at 30 days after the procedure.

### Follow-up

**Figure 4:**
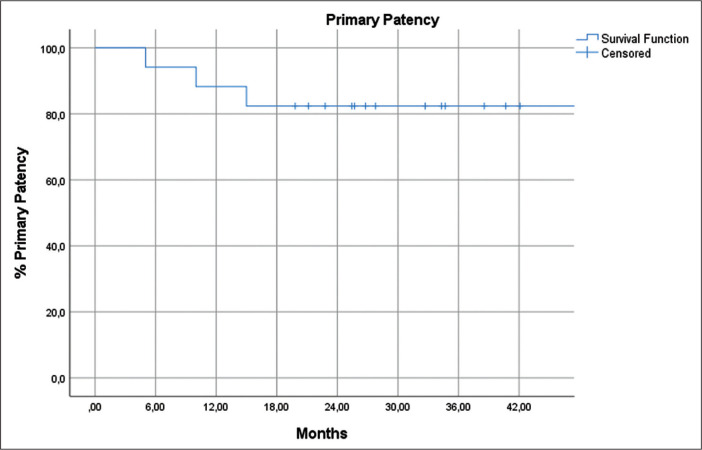
Kaplan-Meier curve for primary patency.

The mean follow-up period was 31.8 ± 8.15 months (range, 19.8-48.2 months). None of the patients died during the follow-up period. There was recurrence of the stricture in three (18.8%) of the 16 patients, occurring within the first two years of follow-up in two of those patients (Table 2). According to the Kaplan-Meier analysis, the 1-, 2-, and 3-year patency rates were 88.2%, 82.4%, and 82.4%, with numbers at risk of 15, 11, and 4, respectively (Figure 4). The estimated mean patency period was 41.5 ± 4.1 months (95% confidence interval: 33.4-49.5).

**Table 2:** Follow-up and patency (n = 16).

Parameter	Time point				
	Immediate	12 months	24 months	36 months	48 months
Follow-up evaluation, n	16	16	16	13	5
Primary patency, n					
After one dilatation session	16	16	16	10	3
After two dilatation sessions	3	2	3	3	2

## DISCUSSION

In the present study, the use of the ABS oversized balloon dilation technique resulted in technical and clinical success in all cases. Those outcomes were achieved with a single balloon dilation session in 13 (81.2%) of the patients. In addition, the clinical outcomes following the application of the ABS oversized balloon dilation technique seem to be durable, as evidenced by the Kaplan-Meier estimated 3-year primary patency rate of 82.4%. Furthermore, clinical recurrence of the stricture was observed in only three cases and occurred within the first year after successful dilation in two of those cases, justifying a stricter follow-up regimen during the first 12 months of follow-up.

The use of oversized balloons for anastomotic biliary-enteric strictures is widespread in everyday clinical practice. However, to our knowledge, this is the first study to investigate a methodology aimed at safely achieving maximal stricture dilation, based on pre-procedural imaging measurements. The ABS oversized balloon dilation technique was also proven to be safe, given that there was only one major complication—dehiscence of the biliary-enteric anastomosis. That complication was likely attributable to the fact that balloon dilation was performed only three months after surgery, when the anastomosis was not fully healed, with minimal fibrous content and greater propensity for rupture and recurrence of the stricture. Therefore, we suggest that such procedures be performed at least six months after the original surgical procedure, if possible, in order to maximize balloon diameter safely, avoid complications, and increase the chance of achieving clinical success after a single session.

Recent studies conducted in Brazil have highlighted the importance of interventional radiology to improving the diagnosis and treatment of abdominal conditions^(8-10)^. Due to the low level of evidence available in the current literature, the treatment of benign biliary strictures remains controversial. Various interventional radiology techniques have been developed over the years in order to optimize clinical outcomes. The two most commonly described methods are balloon dilation and stent insertion^(11-14)^. However, disappointingly low 3-year patency rates have been reported following conventional percutaneous balloon dilation and PTBD placement for the treatment of benign biliary strictures^(15)^. The optimization of immediate and long-term outcomes continues to be a challenge. Different indwelling catheter techniques have emerged, and percutaneous management with a large-bore indwelling catheter has been proposed, as has a dual catheter placement technique^(16,17)^. Park et al.^(18)^ described a long-term indwelling balloon catheter technique for the treatment of benign biliary strictures. That technique consists in placing a pigtail catheter, together with a balloon catheter that is 10-15% larger than the estimated diameter of the duct. Because of the above mentioned procedural benefits, those authors reported that the technique has major advantages in terms of pain management and treatment effectiveness, although it has the disadvantage of the long dwell time of the drainage catheter, which can be uncomfortable for the patient.

Retrievable covered stents have been used to treat benign biliary strictures, with promising results, potentially providing better long-term patency than does conventional balloon dilation^(15,19,20)^. In a retrospective analysis of 66 patients, comparing the use of a retrievable self-expanding metallic stent with that of percutaneous balloon dilation followed by PTBD for the treatment of benign biliary strictures^(15)^, the 3-year primary patency rate was reported to be significantly lower after percutaneous balloon dilation (44% vs. 87%). Kim et al.^(15)^ placed retrievable covered stents in 35 benign biliary strictures, and the stents were electively removed after 0.5-5 months. However, previous studies have evaluated samples with significant heterogeneity, including strictures caused by diverse causes such as intraoperative injury, anastomotic stricture (biliary-enteric and duct-to-duct), chronic cholangitis, pancreatitis, trauma, and sclerosing cholangitis^(1-4,13,14,17,21)^. Because such strictures may differ in location, extent, and pathophysiology, different treatment responses should be expected, limiting the generalizability of the results. In contrast, the present study focused on the management of anastomotic biliary-enteric strictures, which have a specific pathophysiology and anatomical location. In addition, we excluded patients who had previously been treated for benign strictures due to biliary-enteric surgery, which can limit the treatment options, such as additional surgery or endoscopy.

Yun et al. reported that temporary placement of a covered stent provides longer patency than does balloon dilation. In their study, the 3-year primary patency rates were 84.9% for the stent group and 52.8% for the balloon group, stricture recurrence requiring re-intervention being 3.7 times more common in the balloon group^(21)^. Their results are in agreement with those of previous studies using percutaneous temporary covered stenting^(15,19,22)^. These results suggest that stents provide prolonged dilatation, as well as inducing stretching and remodeling of biliary-enteric anastomoses^(23)^. However, the fact that the direct cost of stent placement is higher than is that of balloon angioplasty remains an issue and there have yet to be any large-scale randomized studies comparing the two methods.

Our study has several limitations. Because it was a retrospective analysis, some cases might have been missed and the results are subject to selection bias. In addition, reproducibility of the results is not guaranteed, given the two-center design, and there was no control arm, which would have made it possible to draw comparisons with the outcomes of conventional balloon dilation. Furthermore, a small number of patients were enrolled in our study, which prevented us from drawing any firm conclusions. Specifically, the number at risk at three years of follow-up was < 10, which limits the statistical validity of the estimation of primary patency. The results of this pilot study should be confirmed in larger studies.

In conclusion, our findings indicate that a single-step, oversized balloon dilation approach (the ABS oversized balloon dilation technique) is a feasible treatment for benign anastomotic biliary-enteric strictures. The technique appears to be associated with high rates of long-term clinical success and patency. Large-scale, multicenter, controlled trials are warranted in order to corroborate our findings.
